# Disturbance and the elevation ranges of woody plant species in the mountains of Costa Rica

**DOI:** 10.1002/ece3.5870

**Published:** 2019-11-25

**Authors:** Miguel Muñoz Mazón, Kari Klanderud, Bryan Finegan, Darío Veintimilla, Diego Bermeo, Eduardo Murrieta, Diego Delgado, Douglas Sheil

**Affiliations:** ^1^ Faculty of Environmental Sciences and Natural Resource Management Norwegian University of Life Sciences (NMBU) Ås Norway; ^2^ CATIE‐Centro Agronómico Tropical de Investigación y Enseñanza Turrialba Costa Rica; ^3^ Johann Heinrich von Thünen Institute Federal Research Institute for Rural Areas, Forestry and Fisheries Braunschweig Germany

**Keywords:** biotic interactions, competition, distributions, disturbance, elevation ranges, range boundaries, secondary forest, succession

## Abstract

**Aim:**

To understand how disturbance—here defined as a transient reduction in competition—can shape plant distributions along elevation gradients. Theory suggests that disturbance may increase elevation ranges, especially at the lower range limits, through reduced competitive exclusion. Nevertheless, to date this relationship remains unclear.

**Location:**

Mountains of Costa Rica.

**Methods:**

We compared the elevation range of woody stems over 10 cm dbh (“trees”) observed in plots along two transects spanning a range of elevations in secondary (regrowth) and old‐growth forest (409 and 249 species, respectively). We also estimated these elevation ranges using nationwide data. In addition, we examined the influence of stem size and plot scale basal area (as a measure of competition) on species elevation range limits in the two gradients.

**Results:**

In general, tree species ranges increased with elevation. Species in the secondary forest had broader elevation ranges (100–318 m broader than species in the old‐growth forest; Wilcoxon: *p*‐value <.001). Also, in the secondary transect, individuals with greater diameters had broader elevation ranges than those observed as smaller trees (137 m broader; Kruskal–Wallis: *p*‐value = .03). The lower range limit of species occurred more frequently in plots with lower (vs. higher) basal area than expected by chance in both forest types. We also observed higher elevation upper limits in old growth, but not in secondary forests, with lower (vs. higher) basal area.

**Main conclusion:**

Disturbance relaxes the constraints imposed by competition and extends effective elevation ranges of species, particularly those in secondary forest, to warmer and cooler climates (minimum increase equivalent to about 0.6–1.4°C). Thus, suitable disturbance may assist species persistence under climate change. We believe this is the first study indicating a consistent relation between disturbance and woody plant species distributions along elevation gradients.

## Graphical abstract

Disturbance expands the distribution of woody plants in Costa Rica to higher and lower elevations.

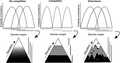

## 1 INTRODUCTION

**Figure 1:**
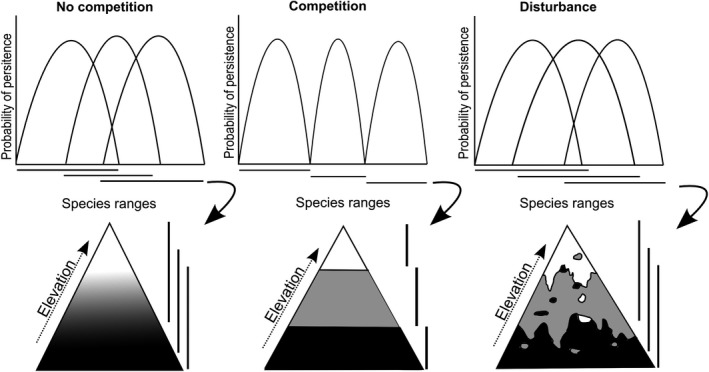
Schematic representation of how competitive hierarchies and disturbance may influence the elevation range of three species. The upper row represents the probability of persistence of three species along a gradient at three different scenarios (no competition, competition, and disturbance). The lower row shows how their ranges would be distributed along an elevation gradient. Under a theoretical scenario of no competition between the three species, their ranges will follow the environmental conditions corresponding to each species' fundamental niche. Competitive interactions would constrain their distributions along elevation gradients, particularly their lower range limits and the realized niche are smaller than the fundamental niche. Disturbance may allow these three species to expand their ranges to upper and lower elevations by altering the already established competitive hierarchies and reducing priority effects so that the realized niche is larger (based on Sheil, 2016)

The idea that tree species distribution patterns in tropical forest are influenced by disturbance is long established—many observations indicate a context‐dependent effect of competition on species persistence along environmental gradients (Budowski, 1965, Van Steenis, 1958 see further examples in Sheil, 2016). Disturbance events, which we define as a transient reduction in competition (resulting from vegetation death or removal), can reduce or slow competition and the resulting exclusion of inferior competitors by freeing up space or resources and by eliminating superior competitors (Sheil, 2016). The role of disturbance in promoting species diversity and (temporary) coexistence is generally accepted at local scales (Fox, 2013; Sheil & Burslem, 2003, 2013), though there is little agreement on how and in what contexts disturbance affects distributions at larger scales (Liang, Duveneck, Gustafson, Serra‐Diaz, & Thompson, 2018; Sheil, 2016; Vayreda, Martinez‐Vilalta, Gracia, Canadell, & Retana, 2016). In theory at least, disturbance might promote the establishment and persistence of species at elevations where they would otherwise be excluded by competitors (e.g., lowlands, see Figure 1). These range expansions imply that some species may be able to persist “in situ” in a warmer future if competition is reduced through a suitable disturbance regime (Johansson, Frisk, Nemomissa, & Hylander, 2018; Sheil, 2016). Predictions of extinctions through contractions in the elevation range of species (e.g., Dirnböck, Essl, & Rabitsch, 2011) highlight the importance of considering disturbance as a conservation tool (Sheil, 2016). However, as far as we know, nobody has quantified the influence of disturbance on species distributions along elevation gradients and estimated how they may influence potential persistence under warmer (or colder) conditions.

Biotic interactions can constrain species distributions and influence range limits (Araújo & Luoto, 2007; Louthan, Doak, & Angert, 2015; Svenning et al., 2014). For example, there is evidence from temperate and boreal locations that competitive exclusion affects the distribution of plant species along elevation gradients (Ettinger & HilleRisLambers, 2017; Olsen, Töpper, Skarpaas, Vandvik, & Klanderud, 2016; Sheil, 2016). While climate often appears to impose a physiological limit to growth and survival at the upper distribution limit (Ettinger, Ford, & HilleRisLambers, 2011; Körner et al., 2016; Normand et al., 2009; Wen, Qin, Leng, Zhu, & Cao, 2018), this is less clear for lower range limits where competition may play a greater role (Choler, Michalet, & Callaway, 2001; Defossez, Courbaud, Lasbouygues, Schiffers, & Kunstler, 2016; HilleRisLambers, Harsch, Ettinger, Ford, & Theobald, 2013; Pellissier et al., 2013). Such relationships between competition and species elevation ranges are potentially important for conservation as distributions may respond to managed disturbances (reviewed in Sheil, 2016). Currently, as disturbance processes are seldom incorporated in formal distribution studies, we know little about such influences and their wider implications.

Managed disturbance offers a potential means to manipulate and extend species range limits, slowing the arrival of more competitive lower elevation species and facilitating species migrations to new areas with suitable environment (Sheil, 2016). As in managed habitats that are already burned, grazed, or mown so as to facilitate certain species and communities over others, practices may be adjusted in various ways. Of course, not all species will respond to disturbance in the same way. The nature, intensity, frequency, scales, and timing of the disturbance regime would be adapted to favor target species. There are likely to be other factors to consider too, for example in a changing climate disturbance will influence replacement processes and influence how species can spread and track both biotic and environmental conditions (Royo & Carson, 2006; Serra‐Diaz, Scheller, Syphard, & Franklin, 2015; Thom, Rammer, & Seidl, 2017). Furthermore, disturbance could accentuate climatic extremes, promoting the persistence of those species more tolerant to drought or frost. There may also be undesirable persistent vegetation states that are favored by certain disturbance processes (Ssali, Moe, & Sheil, 2018). In any case, the use of disturbance would need to be guided by the best available information and would need to be reevaluated locally. Thus, it is crucial to understand how disturbance can modify species range limits so that we know if these options can be adopted for landscape management and conservation.

Plant species distributed over an extended environmental gradient, such as elevation, are typically thought to sort themselves according to a competitive hierarchy in which inferior competitors are displaced to sites supporting less optimal growth (i.e., higher or lower elevations; Sheil, 2016; Shipley & Keddy, 1994; Wisheu, 1998). This reasoning follows from observations that a trade‐off occurs between the competitive ability of a species and its ability to persist under limiting environmental conditions (Michalet et al., 2006; Morin & Chuine, 2006; Salguero‐Gómez et al., 2016). At the same time, this trade‐off implies that species are seldom good competitors over their entire fundamental range (Grime, 1973; Wilson & Keddy, 1986), depending on disturbance events to establish and persist at the less stressful parts of the gradient (Figure 1; Sheil, 2016). Even if several species have similar competitive ability, colonization and priority effects are likely to favor some over others so that few can thrive throughout their fundamental range. In this context, disturbance allows species to expand their range by temporarily removing competitors and priority effects.

Responses to disturbance vary among tree species. Such responses depend on their ability to colonize and to compete (Cadotte, 2007; Connell & Slatyer, 1977; Swaine & Whitmore, 1988). Tree responses thus depend on the competition‐colonization trade‐offs among the species present (Huston & Smith, 1987; Muscarella et al., 2017; Zhang, Qi, & Liu, 2018). In nature, tree species' strategies typically appear scattered along a conceptualized “colonization‐competition axis” representing a broad range of competition and dispersal abilities and strategies (Adler et al., 2014; Salguero‐Gómez et al., 2016). For simplicity, this range of strategies is often simplified and divided into pioneer (disturbance dependent) and nonpioneer species (e.g., Swaine & Whitmore, 1988).

Our objective is to assess and understand the elevation distribution of tree species, specifically their elevation range limits, and how this is influenced by disturbance histories (and inferred competition). We recognized that there will always be alternative explanations for observed ranges and that good replication of gradients would be needed to distill the effects of disturbance and competition from such observations. Nonetheless, we note that two well sample gradients in the same region offers a “proof of concept” and reveals the ability to detect consistent patterns in real data. We examined distributions over two transects located in old‐growth and secondary (regrowth) forests in Costa Rica. Our assumption is that when competitive exclusion restricts species to certain elevations, disturbance may permit them to occur more widely (Figure 1). Thus, we predict that species present in secondary forest will typically have broader elevation ranges than species present in old‐growth forest. Moreover, we predict that within the secondary forest, tree species that include large diameter individuals (vs. those without) will tend to have broader elevation ranges since a greater proportion of these will have established sooner after disturbance and were thus able to establish and grow with less competition. We recognize that some species only represented by small‐diameter stems may also have established early but believe that the comparison between the range sizes of species with different diameters remains a useful statistical generalization as large stems are seldom young. We also evaluated the relationship between species elevation range limits and local (plot defined) basal area with the prediction that range limits will be more frequent in lower versus higher basal area plots (representing lower vs. higher competition).

## 2 METHODS

### 2.1 Study area

We used records of trees, palms, ferns, and lianas with diameters (at 1.3 m, “dbh”) >10 cm from plots placed along two elevation transects established in Costa Rica in secondary and old‐growth forest during 2013. The old‐growth transect comprises 32 0.25 ha plots from 430 to 2,900 m asl along the Atlantic slope of the Talamanca Mountains. The secondary forest comprises 29 0.1 ha plots from 650 to 1,800 m asl and nine 0.25 ha plots from 1,800 to 2,700 m asl along the northern face of the Turrialba Volcano. The height of the mountain ranges where the transects are located is similar (3,324 m asl for the Turrialba Volcano and 3,451 m asl for Talamanca Cordillera transect), with the treeline occurring between 3,000 and 3,200 m asl (B. Finegan & D. Delgado, personal observation). Mean annual temperature varies from 10 to 25 °C along both transects. Precipitation ranges from 2,000 to 5,000 mm per year, peaking at middle elevations (Hijmans, Cameron, Parra, Jones, & Jarvis, 2005). There is a short dry season between January and March (Kappelle, Uffelen, & Cleef, 1995). Although the length of the dry season does not appear to vary along the elevation gradient, the plots at higher elevations typically receive less precipitation than plots at lower elevations during this period (see Appendix S1). Soils at higher elevations tend to be shallower, richer in organic matter and are more often associated with volcanic ash (typically Andosols). At lower elevations, soils are deeper and are typically have a higher proportion of clay (typically Ultisols, CATIE, unpublished data; Veintimilla et al., 2019).

Both transects include Lowland (>700 m. asl), Premontane (700–1,500), Lower montane (1,500–2,200), and Upper montane forest (2,200–2,900; Holdridge, 1987). The lowland old‐growth forest includes many palms such as *Euterpe precatoria* and *Welfia regia* (Veintimilla et al., 2019). With increasing elevation, the forest transitions to montane forest, where lianas disappear, palms are scarce and species with more temperate affinities like the oaks (*Quercus bumeloides* and *Quercus costarricensis*) tend to dominate along with *Podocarpus* spp., *Magnolia* spp., *Ilex* spp., *Drymis granadensis* and *Ocotea* spp (Kappelle, Kennis, & Vries, 1995; Kappelle, Uffelen, et al., 1995). Oaks (*Quercus* spp.) are absent in the secondary forest, though other species with temperate affinities still dominate at higher elevations (e.g., *Viburnum costaricanum*, *Cornus schiedianus*, CATIE, unpublished data; Murrieta, Finegan, Delgado, Villalobos, & Campos, 2007).

The secondary forest had 21–30 years regrowth postagriculture, according to interviews with the landowners (see Murrieta et al., 2007). Land use before abandonment was pastured at higher elevations, and coffee and sugar cane plantations at lower elevations. Unfortunately, we lack detailed information about prior land use; but we assume that forest trees were scarce or absent. We also lack information on subsequent disturbance processes in the regrowth. There may have been some low‐intensity fuelwood harvesting, but we are confident that there were no fires in this landscape. Secondary forests following coffee plantations likely contain some favored shade trees—typically *Erythrina poepiggiana* and *Cordia alliodora* (Florian, Harvey, Finegan, Benjamin, & Soto, 2008). Although the presence of relic trees along the secondary transect remains uncertain, there are only 41 stems with a dbh >60 cm (of 3,338 stems in total).

Plots were located at least 300 m apart and at least 150 m from the forest edge and avoided obvious disturbances such as larger tree fall gaps. Also, all the plots were established >50 m from watercourses and we avoided very steep areas (slopes >100%). The dominant aspect was east‐facing slopes. In each plot, all stems with a diameter at breast height (dbh) >10 cm were measured, identified at least to morphospecies level, and recorded. Records from Costa Rica of all species identified in the field were extracted from the GBIF database on the 24/05/2018. These GBIF records include field observations, herbarium collections, and occurrences reported in the literature.

### 2.2 Analyses

The observed elevation range of each species was calculated as the difference between the maximum and the minimum elevation in which they occur in our own plot data and according to national data (GBIF). National data include occurrences of species in all kinds of vegetation and therefore do not represent old‐growth forest alone. Nevertheless, the comparison between field versus nationwide elevation ranges is still useful to understand the generality of the observed patterns. The midpoint of the distribution is the halfway point between the highest and lowest record for each species. Due to concerns over uneven sampling, we only consider species with ranges of at least 200 m asl. We tested the relationship between range sizes and their elevation midpoint with a Pearson correlation. In order to compare the different ranges sizes of species present at old‐growth versus the ones at secondary forest, we used Wilcoxon test. We also performed these analyses for the species that occur more than twice in both forests and span ranges >200 m asl. For the relationship between range size and dbh, we first divided each dataset into three dbh categories (10–15 cm; 15–30 cm; and >30 cm, note that a species can belong to more than one category). Individuals with a dbh >60 cm (only 41 stems) were excluded from the analysis in the secondary forest since they may represent remnant trees that established before the disturbance. Range sizes of each species were then calculated and compared with Kruskal–Wallis test, and if the *p*‐value <.05 we used a Dunn test to check the difference between each of the categories.

We analyzed plot level basal area versus elevation along each transect with a gamma generalized linear model using an identity link for both old‐growth and secondary forest gradients. Plots with negative residuals were categorized as “Low” basal area and those with positive residuals as “High” basal area. Then, we determined the number of species with an upper or lower range limit within these categories. We also counted stems in each plot as the null probabilities of finding a stem that is the highest or lowest for its species depends on the number of stems observed. To avoid artifacts, we removed implied range‐limit observations in the highest and lowest elevation plots of each gradient from our analysis. Through modeling the basal area changes along the elevation gradient and using the residuals of the regression instead of the real basal area values, we were able to control the effect of any directional change of basal area with elevation on our results. We counted the number of upper and lower range limits in each of the two categories of basal area and compared them to expected values under two different assumptions with a chi‐squared goodness of fit test. The first assumption is that the probability of finding a range limit at Low and High basal area is the same. The second assumption considers that the probability of finding a range limit is greater in plots with more individuals. We calculated this probability by dividing the number of stems in each category of basal area by the total number of stems in both categories per transect. All the analyses were performed with R 3.4.3.0.

## 3 RESULTS

A total of 4,412 and 3,338 stems were recorded and 491 and 275 tree species identified in the old‐growth and secondary transects, respectively. Species with only one record (82 in the old‐growth and 26 in the secondary forest) were excluded. After removing species with ranges ≤200 m, 239 and 105 remained for analyses. The old‐growth and secondary transects share 21 species that occur at multiple sites and have a range over 200 m in both (None of the species are tree ferns, palms or lianas) .

### 3.1 Ranges versus elevation

**Figure 2:**
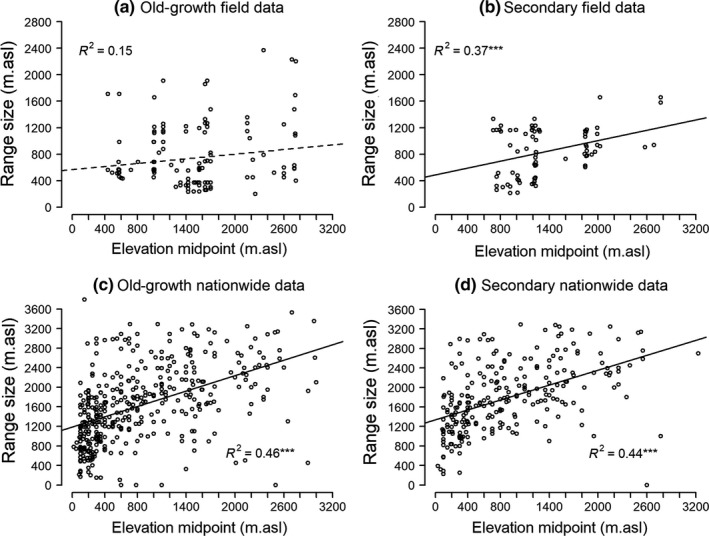
Elevation ranges for tree species from the field data (a) and (b) and from occurrences nationwide (GBIF) (c) and (d) versus elevation midpoint for species with ranges >200 m. asl present in the old‐growth (a and c) and secondary forest (b and d). Linear trend added to aid interpretation. Dashed lines represent nonsignificant relations. *p‐value ≤.05, **p‐value ≤.01, ***p‐value ≤.001

Though plot observations in the old growth are an exception (Figure 2a), we generally observe a significant positive relation between the range size of a species and their elevation midpoint (field data old growth (Pearson correlation coefficient [*r*] = .153, *p* = .075, field data secondary: *r* = .377, *p* < .001, Figure 2a,b); for nationwide occurrences in old growth: *r* = .464, *p* < .001; and in secondary forest: *r* = .445, *p* < .001, Figure 2c,d). Species with narrow elevation ranges (i.e., spanning <500 m) occur through the entire old‐growth elevation gradient (Figure 2a) but appear scarce in secondary forest at higher elevation (Figure 2b).

### 3.2 Elevation ranges in secondary versus old growth

**Figure 3:**
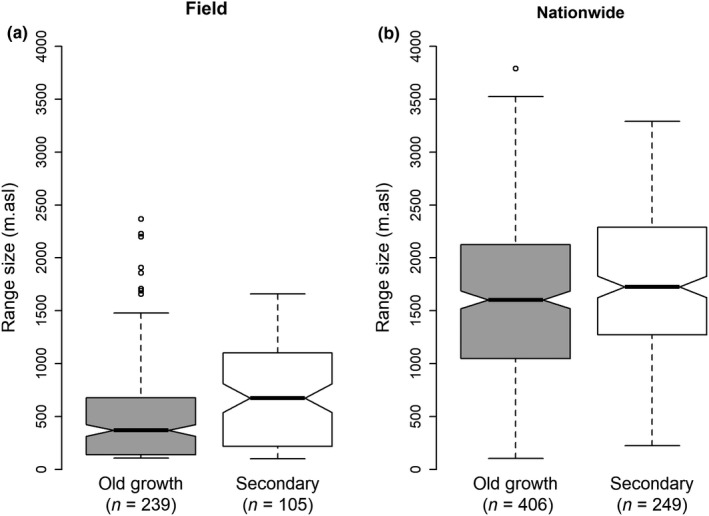
Elevation range sizes for old‐growth and secondary forest species using field (a) and nation level (nationwide) (b) occurrences data from the two elevation gradients

Species in the old‐growth transect generally had narrower elevation ranges than those in the secondary transect. The pattern is apparent using the observed distributions within the sampled ranges (median values 370 and 675 m for old growth vs. secondary, Wilcoxon, *w* = 40,455, *p*‐value = .036) and in the nationwide data (1,603 vs. 1,726 m, *w* = 8,414, *p*‐value <.001, Figure 3a,b), though the differences in range size are greater when using the field data (305 m) than the nationwide data (123 m; Figure 3). When we compare the 21 species with ranges >200 m present in both transects, we find that the median range size is about 200 m greater in the secondary transect though there is considerable variation among observations and the difference is not significant (median range size 650 vs. 851 m for old growth vs. secondary, w = 188, *p*‐value = .4).

### 3.3 Ranges and tree size

**Figure 4:**
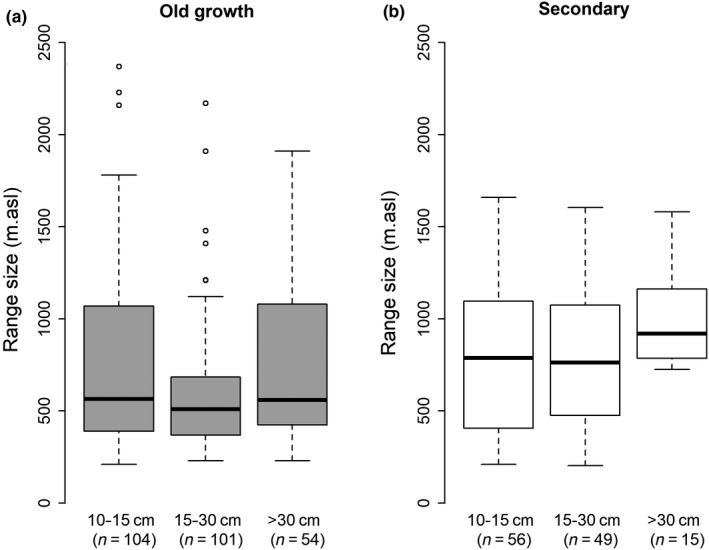
Species elevation range size per diameter class (between parentheses the number of species present at each diameter class) for old‐growth (a) and secondary forest (b)

The observed elevation ranges of species by tree size show no consistent trend in the old‐growth transect (median values 565, 510, 560 m for small, mid, and big sized stems, Kruskal–Wallis w = 1.03, *p*‐value = .668). In the secondary transect, trees bigger than 30 cm dbh have broader distributions (137 m broader, median values 787, 762, 920 m for small, mid, and big sized stems; Kruskal–Wallis w = 7.23, *p*‐value = .03, see Figure 4). All fifteen species observed to reach sizes over 30 cm dbh in the secondary forest are fast‐growing pioneer species (i.e., *Castilla elastica* Sessé ex Cerv., *Cecropia peltata* L., *C. alliodora* [Ruiz & Pav.] Oken, *Croton draco* Schltdl. & Cham, *Hampea apendiculata* [Donn. Sm.] Standl, *Heliocarpus apendiculatus* Turcz., *Inga oerstediana* Benth. ex Seem., *Myrcianthes rhopaloides* [Kunth] McVaugh, *Ocotea austinii* C.K. Allen, *Oreopanax xalapensis* [Kunth] Decne. & Planch, *Symplocos serrulata* Bonpl., *Trema micrantha* [L.] Blume, *Trichospermum grewiifolium* [A. Rich.] Kosterm., and *V. costaricanum* [Oerst.] Hemsl, *Virola koschnyi* Warb.).

### 3.4 Range limits and relative basal area

**Figure 5:**
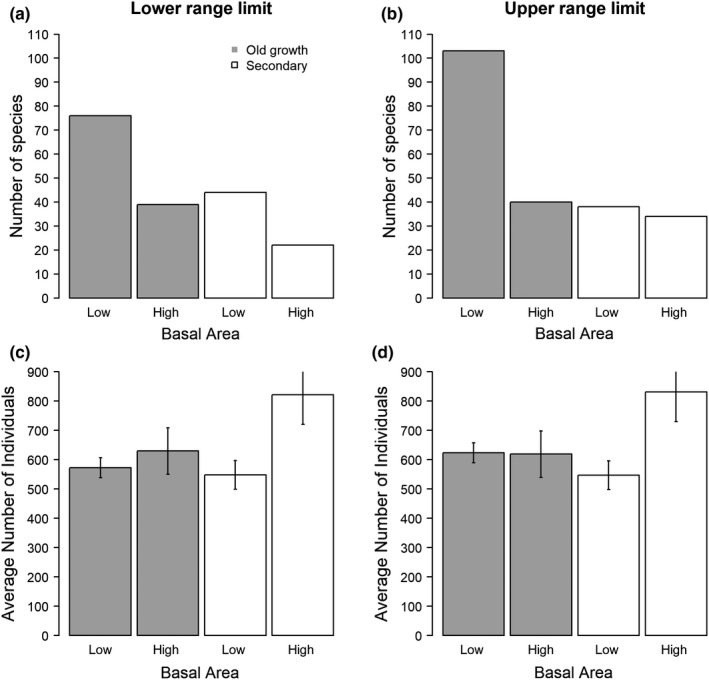
Number of species with their lower (a) and upper (b) elevation range limit observed within plots with a basal area higher (“High”) or lower (“Low”) than predicted by a generalized linear model at that elevation. (c) and (d) show the average number of stems in plots where species have their lower (a and c) and upper (b and d) range limits, respectively

Plot level basal area increases with elevation in both transects (old‐growth forest: coef = 0.006, *SE* = 0.001, *t* = 3.973, *p*‐value <.001; secondary forest: coef = 0.006, *SE* = 0.003, *t* = 2.826, *p*‐value <.001). More species have their lower range limit in plots with below‐average versus above‐average basal areas in both old‐growth (*χ* ^2^ = 11.9, *p*‐value <.001) and secondary transects (*χ* ^2^ = 19.5, *p*‐value <.001, Figure 5). We also observe an excess of highest elevation occurrences in below average basal area sites in old growth (*χ* ^2^ = 15.7, *p*‐value <.001), but this difference decreases in the secondary forest (*χ* ^2^ = 5.1, *p*‐value <.05; and under the assumption of an effect of the stem number *χ* ^2^ = 0.2 *p*‐value = .637).

## 4 DISCUSSION

We found that typical elevation ranges of the species present tended to increase with elevation. Furthermore, species found in the secondary forest exhibited broader elevation ranges than those in old growth. Within the secondary forest, species present as larger stems also tended to possess broader elevation ranges than species present only as smaller stems. We also found that species tended to have both their upper and lower elevation range‐limit occurrences in plots with lower than average basal area at that elevation. This tendency was particularly marked at the lower range limit. Taken together, these results are consistent with our predictions concerning the role of competition constraining (reducing) species elevation ranges and disturbance expanding them (Figure 1). Though there is noise and uncertainty in these data, the implied elevation changes are of the order of hundreds of meters and thus have implications for species occurrence and persistence.

### 4.1 Elevation ranges versus elevation

Increasing species elevation ranges with elevation has been noted previously using herbarium records from Costa Rica (see Stevens, 1992) and the Andes (Feeley & Silman, 2010), but see also (Lieberman, Lieberman, Peralta, & Hartshorn, 1996). What determines these patterns? Geometric constraints play a role—we cannot observe a broad range for species that are restricted to the ends of the observed (or available) gradients. While this limits the observed lower elevation species, it is less evident why we lack narrow range species at high elevations (Figure 2). Ranges at higher elevations may reflect the broad climatic tolerances required to persist at those elevations (Janzen, 1967; Morin & Lechowicz, 2013; Stevens, 1992). In our study, temperature decrease and precipitation seasonality increases with elevation (see Appendices S1 and S2). Trees with a greater tolerance to frost and seasonal drought have been observed to be more widely distributed than less tolerant species (Anderegg & HilleRisLambers, 2016; Esquivel‐Muelbert et al., 2017; Pither, 2003). Nonetheless, the greater investment in tolerance and adaptations required of species able to persist at higher elevation may limit their ability to grow fast and avoid competitors under conditions that do not require such tolerance and adaptations (Koehler, Center, & Cavender‐Bares, 2012; Loehle, 1998). The same trade‐offs are believed to explain the range size of trees in North America (Ettinger & HilleRisLambers, 2017; Morin & Chuine, 2006; Morin & Lechowicz, 2013) and in the Neotropics (Bemmels et al., 2018). Such patterns and trade‐offs are consistent with the presence of a competitive hierarchy.

The presence of narrow‐ranged species along the whole elevation gradient in the old‐growth forest seems to explain the lack of a marked correlation between elevation range size and elevation midpoint in the old‐growth forest (Figure 2). While broad‐range species must possess broad environmental tolerances, narrow‐ranged species may be constrained by narrow environmental tolerances or competition (Ghalambor, Huey, Martin, Tewksbury, & Wang, 2006; Kessler, 2001). We also see that species with narrow elevation ranges become less frequent with increasing elevation in the secondary forest, where competition was temporarily reduced. The ability to establish in previously cleared sites may have released some otherwise narrow range species from competitive restrictions, or the history in these areas may have eliminated them, since open sites can accentuate climatic extremes when compared to closed forest, increasing exposure to drought, frost, and other factors (Rehm & Feeley, 2015a).

### 4.2 Disturbance and elevation ranges

The relation between disturbance and species ranges becomes clearer when comparing old‐growth and secondary forest. The broader elevation range sizes (about 100–318 m, equivalent to about 0.6–1.4°C in temperature) of species in the secondary forest, compared to the species in old‐growth forest, suggest that the initial period of regrowth when the forest started regrowing from open land allowed them to expand their ranges. Many species observed in secondary forest, especially the larger trees, are pioneers with good dispersal that depend on open habitat to establish. Consequently, in a secondary forest, those species that establish first benefit most from the temporary absence of competition, which permits them to expand their ranges beyond what is observed under more intense competition. For instance, we found that species with stems larger than 30 cm dbh in the secondary forest (but omitting any likely relics with a dbh >60 cm), that is, those species that we assume arrived soonest and grew fastest as a result of limited competition, have broader elevation ranges than those with only smaller stems (averaging 137 m or around 0.6 C^0^ broader; Figure 4b). Although the size of a stem does not always reflect when it established, it seems safe to assume that most stems >30 cm dbh in the studied secondary forest represent early arrivals that established when these areas were just abandoned and still open. To our knowledge, only one of the species, *Cordia alliadora*, might plausibly have been transplanted along the elevation gradient, given its use as a shade tree in coffee plantations. Surprisingly, the elevation ranges of the 22 species present in both the old‐growth and secondary transects were not statistically different, although on average these species occurred over a broader elevation range in the secondary forest (200 m broader). This group is composed by a small number of pioneer (e.g., *Cecropia* spp., *Hedyosmum* spp., *Schefflera rodriguesiana*) and late successional species (e.g., *Pouteria reticulata*, *Guarea* spp.). The relatively small sample sizes likely explain the lack of a statistical difference. While dispersal plays some role in the patterns, we have observed (discussed below) it is unlikely to be the sole explanation as it fails to account for the high frequency of range‐limit observations occurring in low basal area sites. While other authors have noted links between species distributions and competitive abilities (see Sheil, 2016), we believe ours is the first to test a theoretical link with disturbance and elevation ranges in natural communities.

Propagule availability limits potential range expansion. Propagules may only be present in a portion of a range where they would otherwise establish and grow. Since we cannot distinguish the influence of propagule availability and dispersal limitation from environmental limits, the difference in elevation range sizes between species in old‐growth and secondary forest provides a minimum estimate of what might be possible if propagule availability were unlimited. This underestimation appears particularly important for species with short localized dispersal and short‐lived seeds. We know for example that many old‐growth species have median dispersal distances of only a few tens of meters (Muller‐Landau, Wright, Calderón, Condit, & Hubbell, 2008) and their seeds lack long‐term viability in the soil (Long et al., 2015; Vázquez‐Yanes & Orozco‐Segovia, 1993). When the climate is changing, we can ask both whether the species can track suitable climates over the landscape (e.g., moving upslope in warming conditions) and also whether they can tolerate the new conditions in situ (Corlett & Westcott, 2013). The first depends on good dispersal, but the second does not. We speculate that in a warmer climate, disturbance may not only facilitate range shifts in species with good dispersal but may also improve the in situ persistence of species with limited dispersal (Sheil, 2016).

### 4.3 Competition and range limits

Competition reduces lower elevation range limits. Species in both old‐growth and secondary forest had their lower range limit in plots with lower (vs. higher) basal area more frequently than expected if competition was irrelevant (Figure 5a). We infer that reduced basal area favors the establishment of species that are otherwise excluded by competition. Other studies have described a similar relation between high tree cover, competition for light, and elevation range limits in shrub and nonwoody plant species at temperate (Nieto‐Lugilde et al., 2015) and tropical ecosystems (Johansson et al., 2018). These studies found that reductions in tree cover helped shrub species to expand their range to lower elevations. Furthermore, observations show that many higher elevation plant species are sometimes observed in disturbed sites at lower elevations (Kappelle, Kennis, et al., 1995; Lovett, 1996; Sheil, 2016; White, 2013). Our observations indicate that many species can establish and persist in lower and warmer conditions if competition is reduced, in this case in the open habitat after land is abandoned, but presumably in any open sites created by disturbance.

Upper range limits appear less clearly influenced by competition than the lower limits. Nonetheless, in the old‐growth observations, disturbance appears to increase the upper range limit with species having their upper elevation range limits in plots with lower (vs. higher) basal area more frequently than otherwise expected (Figure 5b). We again infer competitive displacement given that any species is likely to be less competitive near to their physiological range limits (McGill, 2012). On the other hand, competition has less apparent influence on upper limits in the secondary forest (Figure 5b), suggesting that, if competition remains important, opposing positive interactions may also be present. Positive interactions may protect species from drought stress, frost, and other threats that are more severe in more open locations (Callaway et al., 2002; Maestre, Callaway, Valladares, & Lortie, 2009; Rehm & Feeley, 2015b). Our interpretation is that depending on the nature and vulnerability of the biotic interactions (negative or positive) disturbance can modify these relationships with consequences (positive or negative) for each species' upper range limits.

### 4.4 Methodological limits and lessons

Our comparison of elevation ranges of tree species in secondary and old‐growth forest represents an exceptional opportunity for an initial exploration of how disturbance histories may influence species distributions. From our perspective, it provides a “proof of concept” that these theorized patterns can be detected and explored in these real world data. We acknowledge limitations that should be addressed in future work. Though climatically similar, our two gradients were not perfectly matched and were not replicated at a regional scale: Thus, environmental influences cannot be formally accounted for. For example, we cannot account for the difference in soils while studies elsewhere in the tropics have shown that these can influence species distributions and competition (e.g., Paoli, Curran, & Slik, 2008). Another concern is the influence of sampling effort. The number of stems recorded at each elevation governs the probability of detecting a species. Greater coverage and replication would improve our data and permit broader generalization. Furthermore, we have not examined the many disturbance processes, both natural and human‐made, that impact on these forests. Nonetheless, while these uncertainties matter, and robust statistical inferences require greater replication, we remain confident in our general conclusions as they are not readily explained by artifacts, and match our expectations and more ad hoc observations elsewhere (see Sheil, 2016). Finally, we note that while species occurrence is necessary for persistence, it is not sufficient—further work would be required to clarify if these occurrences at range limits contribute to population maintenance and growth (Pulliam, 1988; Sheil, 2016). We encourage others with suitable data to further explore these relationships.

## 5 CONCLUSION

We have found patterns that appear largely consistent with how we expect competition may influence (limit) the upper and lower distribution of tree species in the mountains of Costa Rica. We also demonstrate that, as previously suggested (Sheil, 2016), under suitable conditions a release from competition that opens up areas for recolonization (as might result from a disturbance event or from the abandonment of previously cleared land) can expand the elevation ranges that result from these competitive influences (Figure 1). This process can increase the elevation ranges for some species, with a consequent increase in their climatic range of occurrence by 100–300 m in elevation or 0.6°C and 1.6°C. These values may be a substantial underestimate due to the confounding influence of dispersal limitation. Under rapid climate change, any means to improve the persistence of species outside their normal climatic range merits consideration. Our evidence suggests that disturbance, by reducing competitive exclusion, can permit at least some species to occur at lower elevations than otherwise. Therefore, disturbance offers a possible means to manage and maintain distributions and possibly improve species persistence in a warmer future, where competitive exclusion may contribute to local and global extinctions. Although our results are best viewed as a proof of concept, rather than a last word, we underline that of the role of disturbance histories is a vital, though often missing, element in understanding and potentially managing species distributions. To develop these approaches into a practical form of conservation management would require further evaluation of the response of particular species to particular forms, scales, and frequencies of disturbance at their range margins.

## CONFLICT OF INTEREST

None declared.

## Data Availability

All the information related to the species composition of the transects is available at CONAGEBIO data repository: http://datos.conagebio.go.cr/collectory/public/showDataResource/dr1. The exact data have been published in the DataverseNO public repository: https://doi.org/10.18710/72JI22
