# Supplementary figures and images for: Disturbance and the elevation ranges of woody plant species in the mountains of Costa Rica
Source: Ecol Evol. 2019 Nov 25;9(24):14330–40. doi: 10.1002/ece3.5870 (PMC6953661; doi:10.1002/ece3.5870)

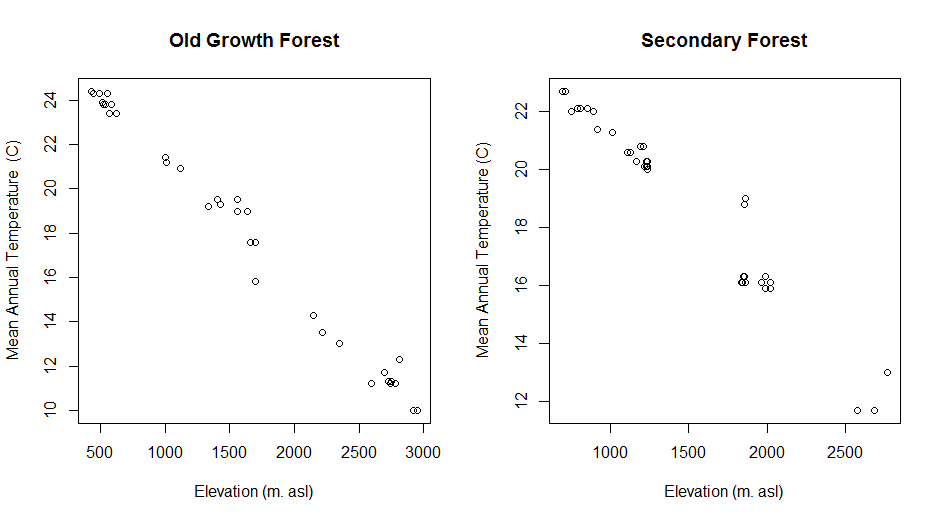

Supplement: Supplementary file 1 [file ECE3-9-14330-s001.tiff]

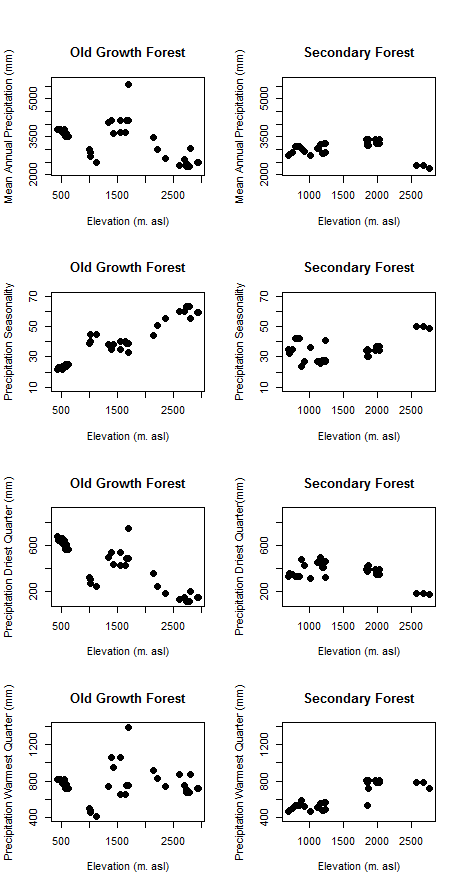

Supplement: Supplementary file 2 [file ECE3-9-14330-s002.tiff]
