# Supplementary material for: Disturbance and the elevation ranges of woody plant species in the mountains of Costa Rica
Source: Ecol Evol. 2019 Nov 25;9(24):14330–40. doi: 10.1002/ece3.5870 (PMC6953661; doi:10.1002/ece3.5870)

**APPENDIX:**

**S1.** Precipitation along both elevation gradients extracted from the WorldClim extrapolated climate map (http://www.worldclim.org/; Hijmans et al., 2005).


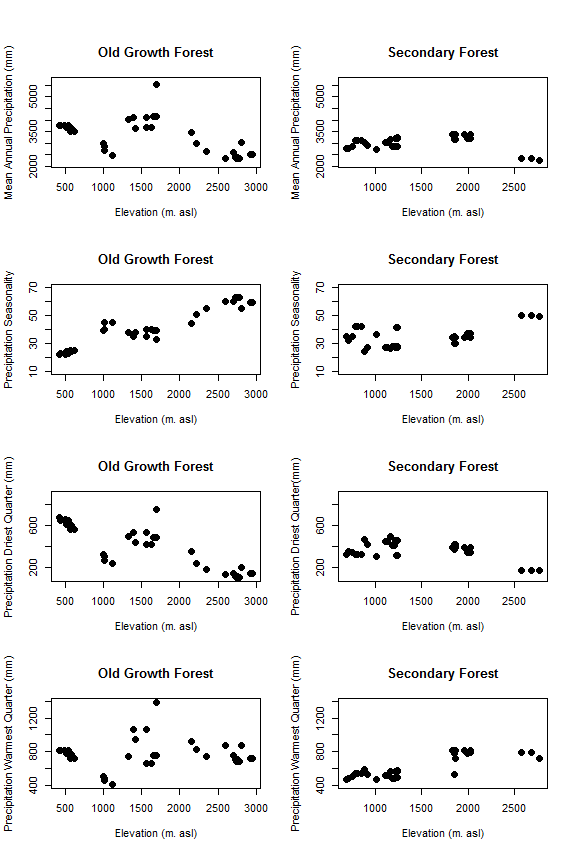


**S2.** Mean Annual Temperature along both gradients extracted from the WorldClim extrapolated climate map (http://www.worldclim.org/; Hijmans et al., 2005).


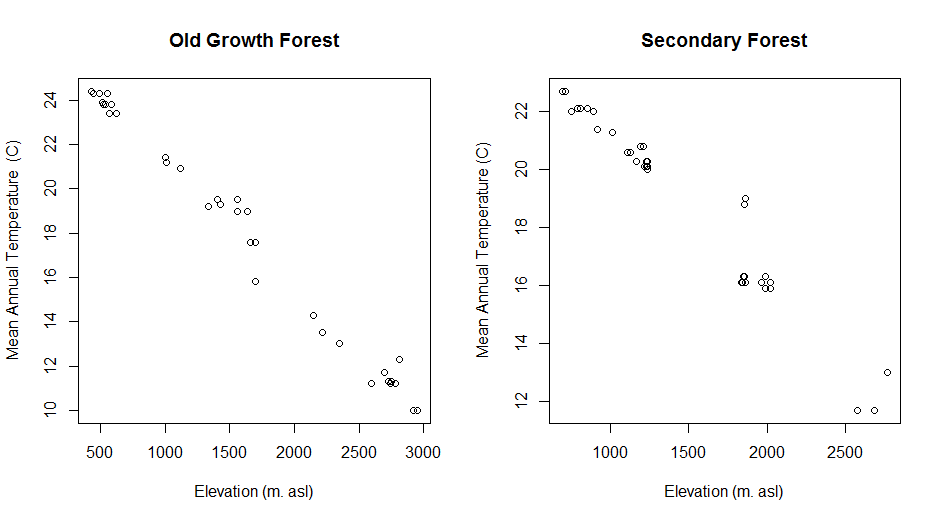

Supplement: Supplementary file 3 [file ECE3-9-14330-s003.docx]
